# Effect of intensive weekend mindfulness-based intervention on BDNF, mitochondria function, and anxiety. A randomized, crossover clinical trial

**DOI:** 10.1016/j.cpnec.2022.100137

**Published:** 2022-04-29

**Authors:** Patama Gomutbutra, Tiam Srikamjak, Ladarat Sapinun, Sukonta Kunaphanh, Nalinee Yingchankul, Nattayaporn Apaijai, Krekwit Shinlapawittayatorn, Rochana Phuackchantuck, Nipon Chattipakorn, Siriporn Chattipakorn

**Affiliations:** a Department of Family Medicine, Faculty of Medicine, Chiang Mai University, Thailand; b The Northern Neuroscience Center, Faculty of Medicine, Chiang Mai University, Thailand; c Department of Occupational Therapy, Faculty of Associated Medicine, Chiang Mai University, Thailand; d The Nursing Service Division, Maharaj Nakorn Chiang Mai Hospital, Thailand; e Neurophysiology Unit, Cardiac Electrophysiology Research and Training Center, Faculty of Medicine, Chiang Mai University, Thailand; f Center of Excellence in Cardiac Electrophysiology Research, Chiang Mai University, Thailand; g Cardiac Electrophysiology Unit, Department of Physiology, Faculty of Medicine, Chiang Mai University, Thailand; h Research Administration Section, Faculty of Medicine, Chiang Mai University Chiang Mai, Thailand; i Department of Oral Biology and Diagnostic Sciences, Faculty of Dentistry, Chiang Mai University, Thailand

**Keywords:** Mindfulness mechanism, BDNF, HRV, Oxidative stress, Anxiety, Clinical neuropsychiatry, Cortisol, Mindfulness Based Flow Practice, BDNF, Brain Derived Neurotrophic Factor, HRV, Heart Rate Variability, MBI, Mindfulness Based Intervention, OXPHOS, Oxidative Phosphorelation, MBFP, Mindfulness Based Flow Practice

## Abstract

**Background:**

The previous metanalysis found that Mind-body intervention (MBI) improves neuropsychologic well-being and may increase brain-derived growth factor (BDNF). BDNF is a neurotrophic factor related to neuroplasticity.

**Objective:**

To evaluate the effect of the short intensive MBI compared to control-relaxation on Site on BDNF and examine if this change is related to mitochondria function or stress-related neurohormonal activity.

**Methods:**

Randomized, controlled, two-period cross-over trial conducted in a medical center in Thailand. Healthy-meditation naive Nurse and Occupational Therapy Students, 23 assigned randomly to MBI, and 24 relaxations at the site for 8 h during the weekend. The wash-out period was three months between the two periods. All volunteers took the blood test for BDNF, mitochondrial oxidative phosphorylation (OXPHOS), Cortisol, and Heart rate variability (HRV) measurement before and Visual Analogue Scale for Anxiety (VAS-A), forward and backward digit span after each period.

**Results:**

A total of 40 participants finished the trials. The cross over trial analysis showed a significant treatment effect between MBI and Relaxation on-site for the mean VAS-A as 9.89 (95% CI 4.81 to 19.47; P = 0.001), serum BDNF as 1.24 (95% CI 0.16 to 2.32; P = 0.04), and OXPHOS complex-1 was decreased 0.41 (95% CI 0.03–0.29 p = 0.03). There were no significant differences for digit span, cortisol, and HRV.

**Conclusion:**

In healthy meditation naïve females, even a short period of MBI may increase serum BDNF and reduce anxiety more than relaxation on-site. The more reduction of OXPHOS complex-1 in the mindfulness group suggests oxidative stress may be a more sensitive indicator than stress-related neurohormonal activity.

## Highlights

• The effects of a short, intensive mindfulness session are scarce.
• Brain-derived growth factor (BDNF) is impacted by mindfulness-based intervention.
• The results indicate that mindfulness practice increased BDNF.
• This can be applied to the promotion of women's health.

## 1 Introduction

Mindfulness-based intervention (MBI) from recent Cochran meta-analysis has a conclusion with evidence that Mindfulness-based intervention is moderately effective in decreasing depression and anxiety symptoms [12]. There was also evidence that MBI has a negligible effect on improving executive function and working memory [29]. Understand basic mechanisms and be able to determine optimum exposure dosage. Several studies showed MBI is associated with both brain structural change as gray matter [20] and functional connectivity change [8]. The most consistent areas that change from MBI include increasing volume and insula area activity related to interoceptive body awareness while decreasing Subgenual anterior cingulate activity related to depressive rumination [3]. These were objective evidence of neural plasticity. However, a study about which immediate biomarker leads to these changes is needed. Several preclinical and clinical studies demonstrated several potential chemical and physiologic biomarkers. These include levels of brain-derived neurotrophic factor (BDNF), mitochondrial impairment, and sympathovagal imbalance measured.

Brain-derived neurotrophic factor (BDNF) is a recognized neuronal growth factor that is important in neuronal survival and regeneration and, therefore, strongly related to neuronal plasticity [16]. Previous studies showed a correlation of brain BDNF to serum BDNF, and serum BDNF also correlates with memory function [17]. Therefore BDNF is interested as a potential mediator underlying the benefit of therapeutic improve age-related and neuropsychiatric related cognitive function [17]. The recent meta-analysis showed that both mindfulness-based exercise and meditation have moderate effects on increasing blood level BDNF[9]. Oxidative phosphorylation (OXPHOS) is a group of enzymes essential mitochondrial function for energy production. Increasing OXPHOS activity leads to increased oxidative stress (reactive oxygen species and reactive nitrogen species). The optimum level of oxidative stress is helpful for immunologic functions. However, excessive oxidative stress could damage mitochondrial itself, leading to insufficient neuronal energy and reducing BDNF transcription [2].

Moreover, oxidative stress is recognized as a mechanism underlying sympathovagal imbalance in several neurological diseases, including anxiety [25]. Although previous studies investigated the mechanism of mindfulness, there are two knowledge gaps. First, most of the evidence conducted in a long formal mindfulness training such as eight weeks-Mindfulness-Based Stress Relaxation (MBSR) and derivatives [14,18,23] months long yoga practice [ [4,19,27]. In contrast, the effect of a short intensive mindfulness session is scared. Second, is there any measurable cellular mechanism that MBI impacts besides BDNF? These questions may motivate meditative naïve people to initiate practice and could be biomarkers used for measuring their physiologic change from MBI. In addition, it needs study methodology to address many unmeasurable confounding factors of individual practitioners, such as the extent to which participants engaged with the practice, complex gene expression as well as a proper control group to mitigate the question of whether MBI provides different effects from simple Relaxation or vacation effect [7]. Therefore, this study's primary objective is to investigate the effect of a weekend short course mindfulness-based training called Mindfulness-Based Flow Practice(MBFP), anxiety symptoms measured by Visual Analogue Anxiety Scale (VAS-A), and working memory measured by forward and backward digit span. The second goal of Hypothetic mechanism-related biomarkers including BDNF, Oxidative stress (cortisol and HRV). The secondary objective is to investigate the effect of MBI on the hypothetic mechanism related to OXPHOS expression or stress-related neurohormonal activity.

### 1.1 Experimental procedure

#### 1.1.1 Trial designs

The study was performed in a single site randomized controlled two-period cross-over design to explore the short-term effect of intensive 8-h mindfulness practice on the BDNF and attention span. A cross-over design was chosen for this study instead of the more traditional randomized, parallel-group design because each participant acted as their control and thus required fewer volunteers. In addition, some of the known disadvantages of the cross-over design, such as a more significant drop-out rate or instability of the volunteer's condition, were not expected in this study because all were healthy people working or studying in a single institute. Furthermore, we try to eliminate a potential carry-over effect by a sufficient wash-out period. Based on a previous study with 35 min of mindfulness intervention that applied wash-out periods 2 h [Håkansson et al., 2016], we assume that our 8 h of intervention may need at least 32 h for the wash-out period. Since a preclinical study showed a half-life of peripheral BDNF was short as 10 min [21], it would not cause period effect. Finally, we brought a volunteer vote to select three mounts' wash-out period in this study. The study had been approved by the Faculty of Medicine Chiang Mai university's research ethics committee (FAM-2561-05328). The study report follows the [CONSORT 2010] extension to randomized cross-over trials[5]. There was a change in the outcome that we did not use depression by HADs because no volunteer has a score above 7.

#### 1.1.2 Participants and randomization

Participants were recruited from the self-enrolled volunteer from promotion during an interdepartmental conference between nursing professionals and occupational therapy school. Therefore, our volunteers comprised nurses, nurse assistants, and occupational therapy students. The criteria included pre-menopause females without serious physical or psychological illnesses (as phase I trial studies healthy volunteers), individuals with atrial fibrillation or on heart devices, and those who are unsure whether they can participate in the trials, which are three months apart.

All participants provided informed consent. The randomization into two groups; group A receives relaxation on-site first, and group B receives MBFP first, was done by computer-based randomization using permuted blocks. The generated random sequence was transferred to the sealed envelope to conceal the randomization till the actual allocation.

#### 1.1.3 Intervention

In both groups, all participants underwent the 8-h MBFP on the weekend- Saturday and Sunday. There were four of 2-h sets of MBFP in total. Those took place on Saturday (9–11am, 2–4pm) and Sunday (8–10am, 1–3pm). Each 2-h MBFP session included 1 h of stretching, 15 min of hypnotic emotional relief, 15 min of meditative breathing, and 15 min of self-massaging. Meanwhile, the relaxation on-site, the participant was assigned. Both groups living in the same resort were similar in both interventions, having the same food and directed not to bring computer or work documents to the resort. During the intervention, they were also not allowed to practice heavy exercise like bicycling gym training.

Baseline data were collected one week before the first period. There were three categories including; 1) demographics: gender, age, race, educational level, employment status.

2) Physiological data: blood pressure, body mass index (BMI), and waist-hip circumference and 3) Neuropsychological data: anxiety levels (VAS-A), depression (PHQ-9), sleep quality (PHQI) were all assessed by using a self-reporting questionnaire. The Montreal Cognitive Assessment (MoCA). The forward and backward digit span tests were used to evaluate cognitive function, administered by a blind research assessor.

#### 1.1.4 Clinical outcome

We applied the computerized visual analog scale to measure state anxiety (VAS-A), which was validated with https://people.socsci.tau.ac.il/mu/anxietytrauma/visual-analog-scale/. These single-item measures in which participants mark their subjective status on a 0–100 visual scale are rapid and straightforward administration and free licensing. This tool showed a high correlation with the gold-standard multi-item inventories of state anxiety, Spielberger's State-Trait Anxiety Inventory (STAI), and sensitivity to rising anxiety response to the social trigger [1]. First, we aim to measure depression measured by PHQ-9 sleep quality (PHQI), but we found this aspect is low in the baseline.

#### 1.1.5 Biomarker outcomes: BDNF, OXPHOS, and cortisol

Blood samples were collected after overnight fasting between 8 and 9 a.m. They were kept in EDTA-coated tubes and clot blood tube. Serum samples were left to clot for 1 h at room temperature and 1 h at 4 °C. Plasma was obtained by centrifugation of the blood at 3000 rpm, four °C for 10 min, and frozen at −80 °C immediately until analysis. A commercially available quantitative sandwich enzyme-linked immunosorbent assay (ELISA) kit analyzed plasma BDNF levels (ab21166, Abcam, United Kingdom). Plasma cortisol level measurement was performed at the central laboratory of Maharaj Hospital, Faculty of Medicine, Chiang Mai University, Chiang Mai, Thailand. Each analysis was done following protocols provided by the test manufacturer. Each parameter was determined simultaneously with the same batch of ELISA kits by a qualified researcher at the molecular biology unit of the Cardiac Electrophysiology Research and Training Center.

OXPHOS protein expression was determined using a Western blot analysis. The peripheral blood mononuclear cells (PBMCs) were extracted by a Ficoll-Hypaque gradient [24]. The protein was lyzed in the extraction buffer and was loaded onto the Sodium Dodecyl Sulfate polyacrylamide gel. Then, the protein was transferred to the nitrocellulose membrane (Amersham Western blotting membrane, GE Life Sciences, MA, USA). Then, the membranes were incubated with anti-OXPHOS cocktail antibody (ab110411, Abcam, United Kingdom) and HRP-conjugated secondary antibody (7074, Cell signaling, MA, United Kingdom). Actin was used as a loading control (sc376421, Santa Cruz, CA, USA). Western blot was developed by the exposure of enhanced chemiluminescence (Clarity Western ECL Substrate, 1705061, Bio-Rad, CA, USA). It was visualized by the chemo doc touching system (Bio-Rad, CA, USA). The densitometric analysis was done using the Image J program (NIH, MD, USA). Their actin normalized the expression of OXPHOS.

#### 1.1.6 Physiologic outcomes: heart rate variability (HRV)

The SEER Light Holter system (GE Healthcare, Milwaukee, WI, USA). An ECG was recorded continuously for 30 min. The computer software MARS software version 7, GE Healthcare, Milwaukee, WI, USA was used to scan for rhythm disturbance and to detect and label each QRS complex. Excessive noise and artifacts were noted, and ectopy was quantified. There are two methods of HRV measurements, including the time domain (i.e., SDNN, RMSDD) and the frequency domain (i.e., LF, HF, LF/HF ratio). Generally, the time domain is appropriate for evaluation for at least 18 h to observe variation between daytime and nighttime. The frequency domain, mainly LF/HF, is more sensitive to change during the monitoring duration as short as 5 min (“Heart rate variability,” 1996). The higher value of time-domain and HF categorized as 'increase HRV' means the parasympathetic tone is over sympathetic tone. In contrast, lower HRV implies that the responsive system in the autonomic nervous system worsens. The time-domain analyses included average heart rate, average R-R intervals (NN), the standard deviation of the R-R intervals over a 24-h period (SDNN), the standard deviation of all 5-min mean R-R intervals (SDANN), average standard deviation of all 5-min R-R intervals (ASDNN), the percentage of R-R intervals with more than 50-ms variation (pNN50), and the square root of mean squared differences of successive R-R intervals (rMSSD). The frequency-domain analyses were done using Fast-Fourier transform analysis using the same analytical software. The obtained frequency-domain indices were the total power (0–0.4 Hz), high-frequency power (HF, 0.15–0.4 Hz) spectral density, low-frequency power (LF, 0.04–0.15 Hz) spectral density, and very-low-frequency power (0.003–0.04 Hz) spectral density. Total power expresses the magnitude of the entire HRV, whereas HF power reflects the parasympathetic tone, and LF power indicates the sympathovagal interactions. These power spectral densities were expressed in absolute units (ms^2^).

The designated physician who operated and fitted the Holter monitor to the patients was blinded to the patients' information. The subjects were fitted with the Holter monitor after blood sample collection at the Holter unit of the Faculty of Medicine, Chiang Mai University. Every participant was assigned to take a blood test, have meals, and sit in the resting area for 30 min before the test.

#### 1.1.7 Sample size

A priori power analysis using G*Power for finding a medium to large effect size (ES = 0.60) difference between two groups based on a previous meta-analysis[9], with α = 0.05 and power of 0.80 for primary analyses, suggested an estimated sample need is 40 persons. Considering drop-out 20%, we recruited 50 volunteers as a minimum sample.

#### 1.1.8 Data analyses

The commercial statistic program, SPSS(version 22.0, IBM Corporation, Chicago, IL), was used for demographic data analysis. The baseline data were obtained independent t-tests or Mann-Whitney test (for continuous variables) and χ2 test or fisher's exact test (for nominal variables). The paired analysis to find the correlation coefficient between baseline and each treatment considers the within-person differences. This report aims to possible future meta-analysis [6].

NCSS version 2021 [11] was used for c2x2 cross-over analysis. There are two steps of cross-over trial analysis, including 1) test for assumption validity including carry-over effects between both study periods and test for period effect and 2) test for the treatment effect. The student's paired *t*-test has performed with data of the first therapy period also included in the multivariable analysis comparison of our data with other studies. The sum of the measured values in the periods for each patient and compared across the two groups by an unpaired *t*-test. When the p-value was more significant than 0.05, there was no significant carry-over effect after the wash-out period.

#### 1.1.9 Missing value and imbalance baseline approach

All analyses were carried out using the intention-to-treat approach, with missing data calculated using the last observation [26]. This method assumes that participants maintain the same value as the last time measured before dropping out. For example, a participant who has measured OXPHOS on period one (before and after intervention) but has not measured on period two imputes the value of after intervention of period one for both before and after the intervention of period two.

Since our trial had a small number of participants, it naturally increases the risk for imbalanced baseline data after randomization. However, our outcome relies on value changing from baseline, not the absolute value, which would mitigate the complications in interpreting and accepting the trial result. In addition, the influence of baseline imbalance to increase chance bias to the outcome have a more significant effect for larger sample sizes than for small [22]. Therefore, we decided not to adjust the baseline variable in the outcome analysis.

#### 1.1.10 Drop-outs and missing values

**Fig. 1:**
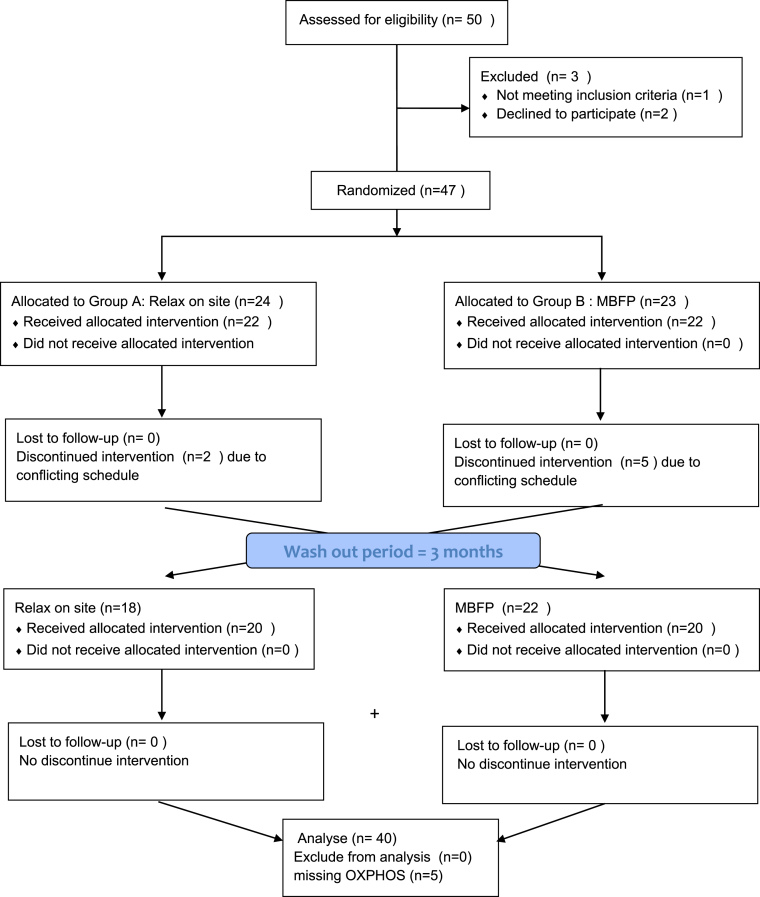
The CONSORT study design. Two-period controlled crossover design. Eache period included 8 h (4 sets) of MBFP or relaxation on site. Three months break was held as for the wash-out between both periods. Before, at crossover and the end of the study, assessments were done. MBFP = Mindfulness based flow practice. Group A = MBFP-relax on site, Group B = relax on site- MBFP.

The 50 volunteers were recruited, but one was excluded due to first diagnosed diabetes from screening fasting blood sugar, and two later changed their minds not participate. Fig. 1 shows a patient flow chart with reasons for decline or drop-out. All of the drops out reasons were due to conflict of schedule (i.e., family issues, social events) not related to interventional complications. Forty volunteers completed both sessions as intended. Twenty-two individuals were assigned group A (relax on-site first), and 18 participants were assigned group B (MBFP first). The drop-out rate, participants who did not receive the final assessment, were 20% missing rate in total. However, there were five missing values for OXPHOS (two for group A and three for group B) due to the wrong blood tube.

## 2 Results

### 2.1 Demographic and baseline values of both groups

The mean age of the volunteers was 23.5 years (20–50 years), and 69% (N = 29) were occupational therapist students. 31% (N = 11) were nurse practitioners. The baseline values of study outcomes, except LH/HF, did not differ significantly between the two study conditions (all p > 0.05, see Table 1). The LF/HF in group A – relaxation on-site first was significantly higher than group B- MBFP first (1.69 vs. 1.34 p = 0.03).

**Table 1:** Baseline demographic and clinical characteristics by sequence: Group A Relaxation on site-MBFP, Group B MBFP – relaxation on site (N = 47).

Factors	Group A (N = 23)	Group B (N = 22)	p-value
	N(%)/Mean ± SD)/Median(IQR)	N(%)/Mean ± SD)/Median(IQR)	
Age (year)	22(21–39)	24.5(20–50)	.923^m^
Occupation			.566^f^
Register nurse (2)	14(56.0)	11(50.0)	
Nursing assistant (0)	1(4.0)	3(13.6)	
University student(1)	10(40.0)	8(36.4)	
Education			.349^f^
Master (3)	1(4.0)	3(13.6)	
Bachelor (2)	9(36.0)	5(22.7)	
University student (1)	14(56.0)	11(50.0)	
High school (0)	1(4.0)	3(13.6)	
Married (1)	3(12.0)	8(36.4)	.057^f^
Neuropsychological test			
MOCA	26.72 ± 1.74	26.36 ± 1.56	.467^t^
VAS-A (anxiety)	65.05 ± 12.76	63.70 ± 12.20	.713^t^
PHQ-9 (depression)	4(2–6)	4(3–6)	.433^m^
PSQI (sleep quality)	7(4–8)	7(5–9)	.555^m^
Digit span Forward	8.40 ± 1.29	7.36 ± 2.36	.076^t^
Digit span Backward	4(3–4)	3.5(3–4)	.924^m^
Biomarkers			
Morning serum glucose (mg/dl)	86.0(83.0–89.0)	86.5(84.0–94.0)	.522^m^
Morning serum cortisol (μg/dl)	8.64(8.16–12.11)	7.86(5.83–11.30)	.077*^m^
Serum BDNF (ng/ml)	6.97(4.09–9.45)	5.74(2.27–9.90)	.765^m^
Oxphos complex I	0.96(0.79–1.17)	1.13(0.87–1.45)	.388^m^
Oxphos complex II	2.03(1.93–2.40)	1.99(1.61–2.47)	.429^m^
Oxphos complex III	1.16(1.06–1.48)	1.11(1.06–1.34)	.258^m^
Oxphos complex IV	1.43(1.11–2.29)	1.39(1.14–1.72)	.790^m^
Oxphos complex V	1.14(1.05–1.64)	1.40(1.05–1.54)	.974^m^
Heart Rate Variability (HRV)			
Low frequency (LF)	25.43 ± 11.95	20.12 ± 6.55	.062^t^
High frequency (HF)	16.50(8.85–22.03)	13.71(10.85–18.51)	.773^m^
**LF/HF ratio**	**1.69 ± 0.53**	**1.38 ± 0.42**	.034*^t^
SDNN	56.84 ± 16.10	52.54 ± 15.32	.356^t^
RMSSD	31(21–40)	27.5(24–38)	.957^m^

t = *t*-test, m = mann-whitney test, f = fisher's exact test.

*p-value ≤ 0.05: statistically significant with null hypothesis the difference of mean equal 0.

** p-value ≤ 0.05: statistically significant with null hypothesis the mean of fatigue group is more than non-fatigue.

mean fatigue score of total 9 ± SD = 3.83 ± 0.92 in fatigue groups, 1.21 ± 0LLL.73 in non-fatigue groups.

### 2.2 The sensitivity analysis and correlation coefficient of baseline and after the first period

The sensitivity analysis for all outcomes from the first cross-over period was done. The paired within-person before and after the intervention, both relaxation on-site for group A or MBFP for group B in the first period, were analyzed.

**Fig. 2:**
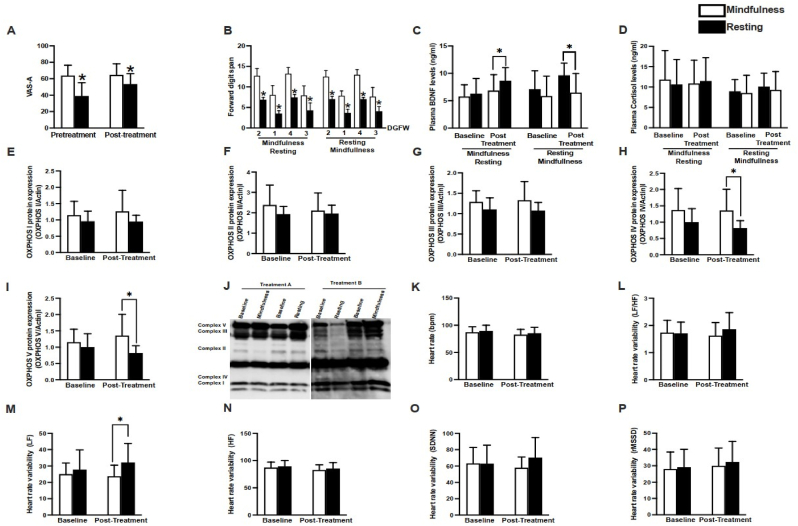
The sensitivity analysis of each outcomes.

The two-sample T-test found that the mean change of VAS-A from baseline in the MBFP group was significantly lower than the relaxation group (13.6, p = 0.01), as shown in Fig. 2A. The score of forwarding digit span was lower in subjects receiving a relaxation protocol than those who received MBFP (Fig. 2B). There were no statistically significant changes from baseline among the two groups for BDNF, as shown in Fig. 2C. The T-test analysis revealed that plasma BDNF levels were higher in the resting group than the mindfulness group, suggesting that an increased plasma BDNF is associated with a lower VAS-A score and forward digit span (Fig. 2A–C). Plasma cortisol levels were not different between groups (Fig. 2D). The protein expressions of OXPHOS complex IV and V were lower in the relaxation group than the MBFP group, while complex I-III protein expressions were not different between groups (Fig. 2E–I). The representative Western blot is shown in Fig. 2J. HRV was used to assess the parasympathovagal balance; our data showed that heart rate was not different between-group (Fig. 2K). HRV analysis demonstrated that although LF was increased in patients receiving relaxation protocol (Figure 2M), LF/HF ratio and HF were not different between groups (Figure 2L, N). Additionally, time-domain analysis of HRV, including SDNN and rMSSD, were not different between groups (Figure 2O, P).The full details are described in Table 2. The correlation was described as the Pearson coefficient correlation (r). For example, VAS-A has a negative correlation between before and after intervention (r = -0.54) for relaxation on-site (r = −0.13).Meanwhile, BDNF has positive correlation (r = 0.69) for relaxation group, (r = 0.60) for MBFP group.

**Table 2:** The sensitivity analysis from the result of the baseline and after intervention either relaxation or MBFP in period one of the cross over trial.

Measurement	Group A (relaxation)			Group B (MBFP)			The difference of changing from baseline: Group B – Group A	
	Baseline Mean (SD)	After Mean (SD)	Correlation R (95% CI)	Baseline Mean (SD)	After Mean (SD)	Correlation R (95% CI)	Mean difference (SE)	P value
Serum BDNF:ng/ml	6.95 (3.58)	7.59 (3.93)	0.69 (0.38–0.86)	6.73 (3.09)	7.25 (2.74)	0.60 (0.22–0.81)	0.88 (0.77)	0.26
Cortisol: mg/dl	12.4 (8.54)	13.43(11.32)	0.12 (−0.14 to0.52)	13.12 (9.10)	13.67 (9.45)	0.15 (−0.23 to 0.65)	0.07 (1.56)	0.96
Oxphos I	1.02 (0.28)	0.85 (0.25)	−0.69 (-0.23 to −0.89)	1.51 (0.89)	1.26 (0.65)	−0.39 (−0.03 to0.69)	- 0.70 (0.46)	0.15
Oxphos II	1.93 (0.38)	1.96 (0.41)	0.71 0.26 to 0.90)	2.78 (1.70)	2.11 (2.08)	- 0.11 (-0.32 to 0.50)	−0.08 (0.24)	0.76
Oxphos III	1.11 (0.28)	1.08 (0.20)	0.13 (0.43–0.62)	1.53(0.76)	1.33(0.46)	−0.17 (-0.26 to 0.54)	−0.17 (0.24)	0.48
Oxphos IV	1.32(0.41)	1.29 (0.20)	−0.68 (−0.19 to 0.88)	1.89(1.02)	1.54(0.77)	−0.32 (-0.12 to 0.64)	−0.26 (0.35)	0.40
Oxphos V	1.00 (0.41)	0.82 (0.22)	0.68 (0.19–0.88)	1.37(0.66)	1.36 (0.64)	0.39 (0.03–0.69)	0.168 (0.22)	0.45
Clinical outcomes								
VAS-A 0–100	71.5(15.6)	56.3(12.35)	−0.13 (−0.56 to-0.01)	64.7(13.5)	32.5(11.45)	−0.54 (−0.75 to 0.12)	−13.76(4.72)	0.01
Forward digit span	7.86 (1.17)	12.50(1.50)	−0.37 (−0.06 to −0.67)	8.06(2.29)	12.72(1.74)	0.43 (0.04–0.73)	−0.24 (0.64)	0.87
Backward digit span	3.68 (0.89)	7.05 (0.65)	0.11 (−0.32 to 0.49)	3.50(0.71)	6.89(0.58)	0.29 (-0.20 to 0.65)	0.02 (0.29)	0.93
Heart rate variability								
LF/HF ratio	1.51(0.51)	1.66(0.51)	0.51 (0.11–0.76)	1.55(0.57)	1.58(0.37)	0.24 -(0.25 to 0.62)	−0.12 (0.17)	0.47
SDNN	50.50(16.20)	48.41(18.79)	0.69 (0.37–0.86)	52.94(12.46)	48.28(21.45)	0.47 (0.01–0.76)	0.86 (4.97)	0.86
RMSSD	27.77(10.84)	25.68(12.45)	0.38 (−0.04 to 0.68)	28.00(9.99)	24.78(11.58)	0.27 (−0.22 to 0.64)	−3.02 (3.42)	0.38

*p-value ≤ 0.05: statistically significant with null hypothesis the difference of mean equal 0 ** p-value ≤ **0.05: statistically significant with null hypothesis the mean of fatigue group is more than non-fatigue** mean fatigue score of total 9 ± SD = 3.83 ± 0.92 in fatigue groups, 1.21 ± 0.73 in non-fatigue groups.

### 2.3 Cross-over analysis

**Fig. 3:**
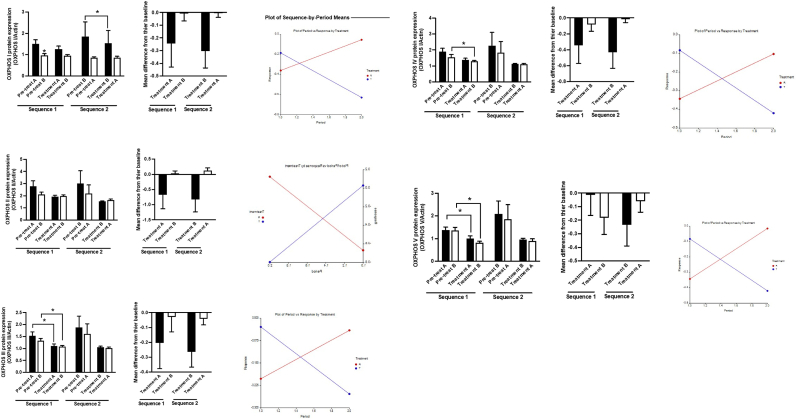
The change of OXPHOS in period one and period two of the cross over trial. Treatment A = relaxation on site, treatment B = MBFP.

All 40 participants completed the cross-over trials. The cross over trial analysis showed a significant treatment effect between MBI and Relaxation on-site for the mean VAS-A as 9.89 (95% CI 4.81 to 19.47; P = 0.001), serum BDNF as 1.24 (95% CI 0.16 to 2.32; P = 0.04), and OXPHOS complex-1 was decreased 0.41 (95% CI 0.03–0.29 p = 0.03). There was no significant treatment effect on other outcomes (Fig. 3). The carry-over effect and period effect of these outcomes were not significant. The full details are described in Table 3. The sequence effect was evaluated by changing the outcomes in both periods and plot of the sequence by period-means shown in Fig. 3. It could be noted that OXPHOS was reduced after both relaxation and MBFP. However, the latter provides more significant changes. There was also a different pattern between group A (relaxation on-site first) and group B (MBFP first), which should be aware of possible unadjustable sequence effects. This observation is supported by the plot of the sequence by period-means which show characteristics of sequence effect [Wang et al., 2016].

**Table 3:** Effect of Mindfulness Base Flow Practice (MBFP) versus Relaxation on BDNF and other biomarkers.

Measurement	Treatment effect (After-Before intervention)				Carry over effect	Period effect	Sequence effect
	MBFP Mean (SE)	Relaxation Mean (SE)	Different effect: MBFP-Relaxation (95%CI)	p-value	p-value	p-value	Examine form the Plot of Sequence-by-Period Means
Biomarker outcome							
Serum BDNF ng/ml (N = 40)	2.16 (0.46)	0.83 (0.40)	1.33 (0.06–2.24)	**0.040**	0.91 no	0.46 no	yes
Cortisol mg/dl (N = 40)	0.007 (0.70)	−0.710 (0.86)	0.71 (−3.47 to 2.05)	0.601	0.46 no	0.43 no	no
Oxphos complex1 ng/ml (N = 35)	−0.41 (−0.15)	−0.21 (0.11)	−0.20 (-0.03 to −0.39)	**0.033**	0.10 no	0.41 no	yes
Oxphos complex2 ng/ml (N = 35)	−0.383 (0.13)	−0.272 (0.11)	−0.11 (−1.02 to 0.79)	0.801	0.97 no	0.02 yes	yes
Oxphos complex3 ng/ml (N = 35)	−0.141 (0.07	−0.122 (0.15)	0.02 (−0.301to 0.261)	0.887	0.82 no	0.47 no	yes
Oxphos Complex4 ng/dl(N = 35)	−0.223 (0.11)	−0.252 (0.14)	−0.03 (−0.466 to 0.409)	0.890	0.82 no	0.17 no	yes
Oxphos Complex5 ng/dl(N = 35)	−0.252 (0.15)	−0.179 (0.12)	−0.08 (−0.514to 0.369)	0.592^t^	0.06 no	0.98 no	yes
Clinical outcome							
VAS-A (N = 35)	−22.54 (7.45)	−12.56 (4.77)	−9.89 (4.81–19.47)	**0.001**	0.60 no	0.75 no	no
Forward digit span (N = 40)	4.95 (0.34)	4.99 (0.39)	−0.03 (−0.65 to 0.59)	0.956^t^	0.04 yes	0.99 no	no
Backward digit span (N = 40)	3.59 (0.37)	3.17 (0.35)	−0.42 (0.05–0.82)	0.060 ^**t**^	0.92 no	0.12 no	no
Heart Rate Variability (HRV)							
LF/HF ratio (N = 40)	0.15 (0.09)	−0.015 (0.09)	0.166 (0.38–0.05)	0.125	0.65 no	0.29 no	yes
SDNN (N = 40)	14.65 (1.94)	12.64 (2.40)	1.98 (−10.17 to 6.21)	0.828	0.40 no	0.00 yes	no
RMSSD (N = 40)	26.21 (3.38)	24.85 (3.11)	1.35 (−12.69 to 9.98)	0.950^t^	0.71 no	0.00 yes	no

t = Shapiro-Wilk test >0.05 the assumption of normal distribution was rejected and therefore t Mann Whitney rank test was used instead paired-T test.

## 3 Discussion

This study demonstrated that 8 h of intensive mindfulness practice versus relaxation on-site in mediation naive is more satisfying in reducing anxiety measured by VAS-A. A day after the practice, the physiologic change induces significant increases in serum BDNF, reducing OXPHOS complex I. Meanwhile, there is no significant difference in working memory tested by digit span, cortisol, and HRV. Although not wholly compatible with our hypothesis, this result gives insight that even a short intensive mindfulness practice might provide observable changes to some biomarkers. The strength of our study is that the mediator and outcome variables were not only self-report questionnaires, which could have an inevitable bias due to the lack of double-blinded method and social desirability effects [Podsakoff_et_al_2003]. Furthermore, we illustrate the correlation coefficient of each outcome data from the first period of cross-over that could be used for metanalysis.

To our knowledge, this is the first cross-over trial study investigating mindfulness's effect to investigate both BDNF and OXPHOS together. The result indicated that mindfulness practice increased BDNF support previous meta-analysis [9] and added that the change could last for a day after completing the practice. In addition, the reduction of OXPHOS was never directly described in a published mindfulness study. The close evidence supports this finding in the study on regular yoga practitioners consistently showing signs of significant reductions in oxygen consumption during regular physical activity [28].

It would be valuable to note that although the concept of stress-inducing inflammation and generating oxidative stress was proposed to be pathophysiology underly linking mental health and physical health [13], the recent systematic review found that MBI does not affect CRP and cortisol significantly [23]. This study may imply that MBI has no effect on inflammatory cytokine in non-serious illness persons. Therefore, oxidative stress might be a sensitive representative of well-being rather than serum cortisol or inflammatory biomarkers.

We did not detect a significant treatment effect on HRV outcomes. Time-domain of HRV would be less sensitive when obtained from short-term recordings. Nevertheless, the frequency domain, particularly LF/HF, should be sensitive enough to detect if there was a trend of changing sympathovagal balance. This finding contradicts what the previous study showed an intensive 10 days vipassana meditation significantly increases HF during 5 min practice compared to the relaxation period [15]. However, it cannot be directly compared since our study aimed to evaluate the 'residual' physiologic change after the practice rather than the change during practice as Krygier et al.

### 3.1 Limitations

There are two significant aspects of potential limitations that made this study result should be interpreted with caution. First, the general limitation of cross-over trial design includes the drop out before finishing the second period, potential carry-over effect, period effect, and sequence effect. Second, The limitation specific to our study include the missing data for OXPHOS.

Our study had a relatively high drop-out rate (7 from 47 or 16%). Anyway, dropping out was not related to the serious adverse event from intervention. Although, more drop-out volunteers received the MBFP in period one but did not participate in the relaxation on-site in period two. It may not be suitable to be implied that these volunteers avoid getting either MBFP or relaxation on site. The missing values managed by the Last Observation Carried Forward: no need to assume worst case or best case for drop out the case. However, this approach would increase the carry-over effect to the cross-over study design and may ignore the natural change of some biomarkers. Regarding the carry-over effect and period effect, we perform the statistical analysis which showed no significant impact on BDNF and OXPHOS. However, we found a remarkable sequential effect evaluate from the graph.

The missing OXPHOS in five participants for the second period of crossing over may reduce the robustness of our finding. However, we did the sensitivity analysis after period one, which showed the tendency of OXPHOS changing if we did the parallel trial instead.

### 3.2 Generalizability and future research

As the heterogenicity nature of participant, intervention, outcome measurement method of mindfulness study, the generalizability of this study would have some limitations. However, it would apply to women's health promotion. Since our participants were healthy-meditate naïve working-age females, this group is the most prevalent anxiety disorder[30]. The MBFP is a simple mindfulness practice combination of yoga and meditation which able to conduct without an exceptional trainer. The BDNF and OXPHOS were relatively feasible blood tests that may be considered included in the further replicated studies.

Future research should examine the online platform of MBFP, which would help reduce the problem of conflicting schedules to attend practice on-site and consistent with social distancing policy under the COVID-19 pandemic.

## Protocol and registration

This trial was registered on the Thailand clinical trial registry: TCTR20180313001 (https://www.thaiclinicaltrials.org) and the Clinical Trials Registry – India no 053485. The MOCA test was done by one certified examiner (THGOMPA 1336461).

## Declaration of competing interest

This research did not receive any specific grant from funding agencies in the public, commercial, or not-for-profit sectors.
