# Corrigendum to “Effect of intensive weekend mindfulness-based intervention on BDNF, mitochondria function, and anxiety. A randomized, crossover clinical trial” [Compr. Psychoneuroendocrinol. (2022) 100137]

**DOI:** 10.1016/j.cpnec.2022.100141

**Published:** 2022-08-01

**Authors:** Patama Gomutbutra, Tiam Srikhamjak, Ladarat Sapinun, Sukonta Kunapun, Nalinee Yingchankul, Nattayaporn Apaijai, Krekwit Shinlapawittayatorn, Rochana Phuackchantuck, Nipon Chattipakorn, Siriporn Chattipakorn

**Affiliations:** a Department of Family Medicine, Faculty of Medicine, Chiang Mai University, Thailand; b The Northern Neuroscience Center, Faculty of Medicine, Chiang Mai University, Thailand; c Department of Occupational Therapy, Faculty of Associated Medicine, Chiang Mai University, Thailand; d The Nursing Service Division, Maharaj Nakorn Chiang Mai Hospital, Thailand; e Neurophysiology Unit, Cardiac Electrophysiology Research and Training Center, Faculty of Medicine, Chiang Mai University, Thailand; f Center of Excellence in Cardiac Electrophysiology Research, Chiang Mai University, Thailand; g Cardiac Electrophysiology Unit, Department of Physiology, Faculty of Medicine, Chiang Mai University, Thailand; h Research Administration Section, Faculty of Medicine, Chiang Mai University Chiang Mai, Thailand; i Department of Oral Biology and Diagnostic Sciences, Faculty of Dentistry, Chiang Mai University, Thailand

The authors regret < add the supplementary file which is the handout of MBFP intervention after the accepted for publication. Therefore this would be our research team responsibility and not that of the Editor. Here is the link to the supplement material https://osf.io/2eskz. DOI 10.17605/OSF.IO/SWV6K>.<add the grants : the Senior Research Scholar Award from the National Research Council of Thailand (to SCC); the NSTDA Research Chair Grant from the National Science and Technology Development Agency Thailand (to NC); and the Chiang Mai University Center of Excellence Award (to NC)>

The authors would like to apologise for any inconvenience caused.

